# Cardiac Critical Care: The Evolution of a Novel Subspecialty

**DOI:** 10.14797/mdcvj.1092

**Published:** 2022-06-03

**Authors:** Ann Gage, Andrew Higgins, Ran Lee

**Affiliations:** 1 Centennial Medical Center, Nashville, Tennessee, US; 2 Cleveland Clinic, Cleveland, Ohio, US

**Keywords:** critical care cardiology, cardiac critical care, cardiogenic shock, cardiac intensive care unit, acute cardiovascular care

## Abstract

Driven by evolving patient demographics and disease burdens over the past several decades, the demands placed on the cardiac intensive care unit have steadily increased. Originally born out of the need for post-infarction arrhythmia monitoring, the modern cardiac intensive care space is now encountering progressively more complex patients with multisystem organ failure and, increasingly, complex mechanical circulatory support. This complexity has fueled a demand for specifically trained cardiac intensivists, and many different training pathways have emerged nationwide. In this article, we provide an overview of the evolution, landscape, training, and future of the subspecialty of cardiac critical care.

## From Coronary Care Unit to Cardiac Intensive Care Unit

Intensive care for the critically ill cardiac patient was first provided in a dedicated coronary care unit (CCU), designed for the rapid recognition and resuscitation of arrhythmias. In the 1940s, arrhythmias accounted for roughly 40% of deaths within the first week after an acute myocardial infarction (MI). These deaths were not due to irreversible cardiac damage, and patients who received prompt recognition of their arrhythmia and subsequent treatment had a significantly higher chance of surviving to meaningful recovery. National attention was further drawn to MI and post-infarct care after President Eisenhower’s widely publicized heart attack in 1955 and his subsequent prolonged hospital stay at a prototypical coronary care unit in Aurora, Colorado.^1^

The desire to provide rapid and life-saving care to such patients led to the creation of the coronary care unit, which housed these patients in a segregated geographical space.^2^ As noted by Thorn et al. in 1967, “No decisive measures are available at present which alter the inexorable course of overwhelming shock or unyielding pulmonary edema, the result of pump failure from extensive necrosis of heart muscle. The coronary care unit is therefore equipped, organized and oriented for the treatment of disorders in rhythm, with special focus on resuscitation of patients experiencing fatal arrhythmias.” There was widespread adoption of the coronary care unit after Killip and Kimball reported a 19% reduction in mortality for acute MI patients without cardiogenic shock who were treated in a CCU.^3^

The CCU from the 1960s has gradually evolved into the modern cardiac intensive care unit (CICU). As noted by many authors, the transformation from the CCU to the CICU is not merely semantic; rather, it reflects changing demographics in the population served within these ICUs—namely, a decreasing number of patients with ST-elevation MI (STEMI) and an increasing burden of cardiogenic shock, heart failure, and primary noncardiac diagnoses.^4,5^ In a retrospective analysis of 29,275 patients admitted to the CICU at Duke University Medical Center, Katz et al. noted a decrease in the proportion of STEMI admissions from approximately 40% of all ICU admissions in 1989 to roughly 20% in 2006.^6^ Similar findings were noted in a multicenter study of academic programs in New York, where only 26.3% of all CCU admissions in 2011 were STEMI patients.^7^ Coincident with the decrease in CICU admissions related to coronary artery disease, Sinha et al. examined 3.4 million CICU hospitalizations from 2003 to 2013 and noted a rise in admissions for noncardiac primary diagnoses from 38.0% to 51.7%.^5^

In a multicenter retrospective analysis conducted by the Critical Care Cardiology Trials Network studying 3,049 admissions to CICUs between September 2017 and 2018, 26.7% of patients had respiratory insufficiency and 21.1% had shock, approximately one third of which were mixed cardiogenic and noncardiogenic shock. Additionally, 21.4% received invasive mechanical ventilation, and mechanical circulatory support was used in 9.5%.^8^ There also has been a significant rise in the prevalence of acute renal failure and sepsis in CICU patients.^6^ These data suggest that the modern CICU provides care for patients with cardiovascular conditions presenting with acute noncardiac illness as frequently as those with primary cardiovascular diagnoses, and they bring into question the best form of training for providers working in the CICU.

## Cardiac Critical Care as a Subspecialty of Cardiovascular Medicine

**Figure 1:**
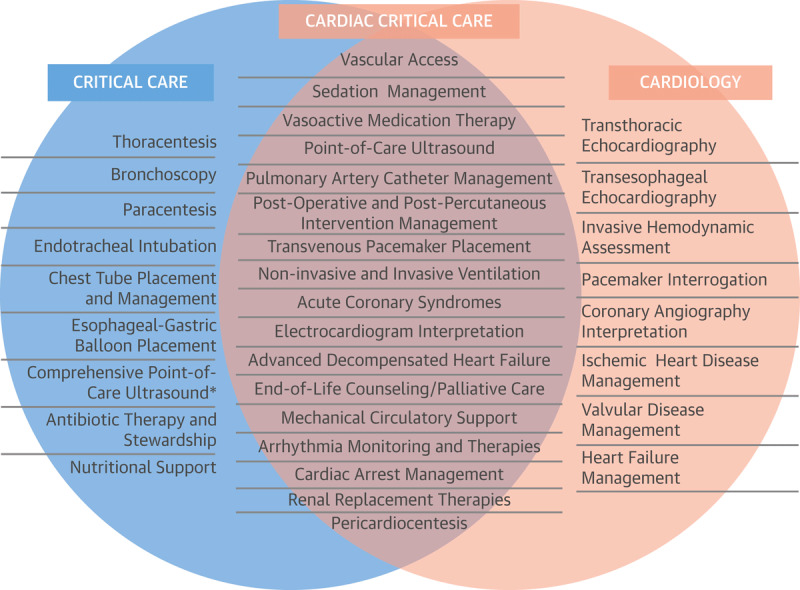
Knowledge and skills for critical care, general cardiology, and cardiac critical care.^9^ Reprinted with permission from Elsevier, Inc. *Lung, abdominal, lower extremity venous ultrasound.

If changing disease states, comorbidities, and patient demographics are to be matched with ideal physician training, CICU providers should possess general cardiology training combined with additional expertise in managing acute respiratory failure, renal replacement therapy, and mechanical circulatory support. In addition, they should be knowledgeable about preventive measures for increasing safety and quality of care in the ICU, such as avoidance of ventilator-associated pneumonia, central-line–associated bloodstream infections, catheter-associated urinary tract infections, and delirium. Furthermore, a robust procedural skillset is necessary, spanning the use of noninvasive and invasive hemodynamic monitoring, vascular access, extravascular procedures, and airway techniques (***Figure 1***).^8,9^ Notably, in a 2011 survey of critical care trainees, participants reported lower confidence in management of cardiovascular versus noncardiovascular diseases.^10^

The aforementioned trends laid the foundation for cardiac critical care as a subspecialty and led to the development of formalized cardiac critical care training pathways.

## Training Pathways for the Modern CICU

Current training pathways for working in a modern CICU are in flux and vary widely from center to center. The current pathway awards dual certification in both cardiovascular medicine and critical care medicine. The American Board of Internal Medicine recognizes a dual subspecialty training pathway consisting of 4 years of fellowship with a minimum of 30 months of clinical training, 6 months of which must be completed in medical critical care units within a critical care training program accredited by the Accreditation Council for Medical Graduate Education (ACGME).^11^ In practice, trainees complete 1 year of critical care medicine through an ACGME-accredited fellowship before or after completion of a 3-year cardiovascular medicine fellowship. This training program builds on the usual cardiovascular medicine skillset with the addition of competencies in airway management, endotracheal intubation, ventilator management, noninvasive ventilation, insertion and management of chest tubes, intravascular procedures, renal replacement therapy, bronchoscopy, nutrition support, and delirium management. Although multiple adaptations to this training pathway have been proposed, none has been adopted by governing bodies or the American Board of Internal Medicine.^12,13,14^

Several variations on this pathway have been pursued by individual cardiac intensivists nationally. Some have elected to pursue a second year of dedicated critical care, allowing more time to hone their airway, bronchoscopy, and pleural skillsets, while others have coupled a dedicated year with a separate year of subspecialty cardiology training such as interventional cardiology, dedicated echocardiography/imaging training, or advanced heart failure and transplant cardiology. Although this does offer the potential for synergy between the critical care and subspecialty skillsets, it also confers the significant opportunity cost of another year of training, as alluded to recently by Carnicelli and colleagues.^12^

There is currently a limited workforce of physicians trained in both cardiovascular disease and critical care,^15^ which limits the expansion of such a staffing model into smaller, community-based hospitals; however, it is likely the appropriate operations model for large tertiary and quaternary care centers. Ultimately, these constraints have led to a hub-and-spoke model for CICUs similar to that developed for cardiogenic shock or high-risk percutaneous coronary intervention, where a hub institution with a trained cardiac intensivist provides a higher level of care for a regional network of hospitals. Such designated Level 1 CICUs are best equipped with cardiac intensivist leadership and input and can provide a wide range of therapies, including but not limited to temporary mechanical circulatory support, invasive mechanical ventilation, and cardiothoracic surgery or interventional procedural services.^13^

## Cardiac Critical Care Improves Outcomes

Since the 1990s, data consistently demonstrate superior clinical outcomes in closed ICUs and in units where patient care is provided by a dedicated critical care provider.^16,17^ In a meta-analysis of 13 studies, a dedicated intensivist was associated with a 39% lower ICU mortality rate (RR 0.61; 95% CI, 0.50-0.75) and 29% lower hospital mortality (RR 0.71; 95% CI, 0.62-0.82) as well as reduced ICU and hospital length of stay.^16^ In a 2012 Scientific Statement on the Evolution of Critical Care Cardiology, the writing group found “the evidence supports a closed structure with staffing by dedicated cardiac intensivists as a preferred approach for the advanced CICU.”^13^

Until recently, data supporting improvement in clinical outcomes for closed ICUs and for ICUs with dedicated critical care providers existed only for general medical and surgical ICUs. However, in 2016, Na et al. demonstrated that the presence of a dedicated cardiac intensivist was associated with a reduction in CICU and in-hospital mortality rates in patients with cardiovascular disease who required critical care.^18^ Subsequently, in a single-center, retrospective study of nearly 4,000 admissions to Yale New Haven Medical Hospital, there was an association between lower in-hospital and CICU mortality after their ICU was transitioned to a closed CICU.^19^ This data informs the discussion of an ideal organizational structure for the CICU and lends support to the need for CICUs staffed by trained cardiac intensivists.

Improvements in outcomes seen in the setting of a closed ICU and/or dedicated intensivist are likely multifactorial. It is postulated that this may be due to increased use of evidence-based protocols,^20^ rapid recognition of life-threatening conditions, attention to patient safety, full attention and presence of an ICU physician,^21^ and/or reduced healthcare-associated infections.^22^

## Research in the Cardiac ICU

Although the adult critical care medicine setting has led to landmark clinical trial data, large prospective clinical trials focused on cardiac critical care patients—in the backdrop of diseases such as acute respiratory distress syndrome (ARDS) and the ongoing COVID-19 pandemic—have been lacking despite the desperate need for rigorous study to inform practice patterns and clinical guidelines. Cardiogenic shock, targeted temperature management for out-of-hospital cardiac arrest, ideal patient selection, and utilization of temporary mechanical circulatory support platforms are some additional areas where knowledge gaps persist. The changing landscape of modern cardiac intensive care offers exciting platforms and opportunities to leverage data and expertise across multiple networks in support of advancing science. To date, this has best been exemplified by the creation of a Critical Care Cardiology Trials Network (C3TN), a multicenter network initially comprised of 16 advanced tertiary CICUs in the United States.^8^ Retrospective analyses from C3TN have examined contemporary insights of cardiogenic shock,^23^ patterns of temporary mechanical circulatory support,^24^ and the comparative use of cardiogenic shock teams in cardiac ICUs.^25^ Prospective trials from networks such as C3TN, in addition to ongoing registry data from others such as the Cardiogenic Shock Working Group offer an exciting future for the study of critically ill cardiac intensive care patients. Combining this infrastructure with highly trained and motivated individuals with mutual backgrounds in critical care and cardiovascular medicine are key elements in advancing scientific knowledge.

## The Future of Cardiac Critical Care

Cardiac critical care is a field in maturation driven by changing demographics of the CICU population and the evolution of cardiovascular care, from peri-infarct care to the treatment of both acute and chronic cardiovascular conditions requiring advanced treatment modalities.

Cardiac critical care is a subspecialty born of the necessity not just to stabilize this patient population but to recognize and address the gaps in the way we provide acute cardiovascular care. As interventional cardiology, structural heart disease, electrophysiology, imaging, and advanced heart failure and transplantation cardiology continue to offer cutting-edge treatments that alter the natural history of acute and chronic cardiovascular diseases, cardiac critical care will continue to provide collaborative care within and among these disciplines to optimize and advance outcomes for critically ill patients with these complex cardiovascular diseases.

## Key Points

- Originally conceived as a post-myocardial infarction electrocardiographic monitoring unit, the cardiac intensive care unit (CICU) has dramatically evolved over the past several decades.
- The modern CICU patient is more acutely ill with more comorbidities than their counterpart from decades prior.
- Training the cardiac intensivist requires cardiovascular medicine training as well as additional competency in critical care medicine. While many programs have developed their own pathways to achieving this skillset, there is currently no consensus on the most appropriate way to educate cardiac critical care trainees.
- The future of cardiac critical care likely includes increased consensus in training models, expansion of the cardiac critical care workforce, increased accessibility of cardiac critical care to acutely ill cardiovascular patients, and the growth of acute cardiovascular care research.
